# Emergence Delirium With Post-traumatic Stress Disorder Among Military Veterans

**DOI:** 10.7759/cureus.921

**Published:** 2016-12-08

**Authors:** Son Nguyen, Mila Pak, Daniel Paoli, Donna F Neff

**Affiliations:** 1 Orlando VA Medical Center, UCF College of Medicine; 2 Anesthesiology, Orlando VA Medical Center; 3 Courtesy Professor, UCF College of Nursing; 4 Associate Dean for Nursing Research, UCF College of Nursing

**Keywords:** emergence delirium (ed), military veterans, post-traumatic stress disorder (ptsd)

## Abstract

The clinical characteristics of emergence delirium (ED) associated with post-traumatic stress disorder (PTSD) among military veterans encompass transient agitation, restlessness, disorientation, and violent verbal and physical behaviors due to re-experiencing of PTSD-related incidents. Two cases of ED after general anesthesia associated with PTSD are presented. Different anesthesia methods were applied for the two cases. A traditional medical approach appeared not to prevent the incidence of ED. In the second case, dexmedetomidine infusion along with verbal coaching was effective in preventing ED for a veteran known to have “wild wake-ups” with prior anesthetics. Further clinical studies in effectively utilizing dexmedetomidine in this population with PTSD at high risk for ED are warranted.

## Introduction

Emergence delirium (ED) is characterized as an altered mental perception that may include confusion, disorientation, illusion, hallucination, and momentary agitation. The etiology of postoperative delirium is known to be multifactorial. Many drugs and drug categories including propofol, ketamine, benzodiazepines, neuroleptics, hallucinogens, anticholinergics, opiates, barbiturates, and volatile anesthetic gases have been implicated. Other clinical studies have identified perioperative physiologic changes such as older age, preexisting comorbidities, and orthopedic surgeries as having a role in ED. To date, though, only limited studies have looked at the prevalence of ED among military veterans diagnosed with PTSD. We present two case reports identifying the unique characteristics of ED in military veterans with PTSD.

## Case presentation

Waived informed consent for a publication of this case report was obtained by a research and public affair department of the Veteran Health Administration system due to no identifiable patient information.

### Case one

A 58-year-old male was admitted for an open reduction internal fixation of his right distal radius fracture. His medical history was significant for impaired glucose tolerance, hypertension, dyslipidemia, obstructive sleep apnea, and asthma. The patient was also diagnosed with PTSD, anxiety, depression, and polysubstance dependency. He reported “wild wake-ups” on awakening from previous anesthetics. He and his wife described episodes of agitation, aggressiveness, and emotional lability manifested as inconsolable crying.

Following placement of American Society of Anesthesiology standard monitors, the patient was preoxygenated with 100% oxygen. He was induced with 100 mg of lidocaine, 200 mg of propofol, and 100 mcg of fentanyl given intravenously. A size four laryngeal mask airway was inserted easily. Anesthesia was maintained with sevoflurane at two to 2.3% in an air/oxygen mixture with a fractional inspired oxygen concentration of 0.56. The patient received 500 mcg of fentanyl and 1 mg of hydromorphone in divided doses during the one hour and 40 minutes of the procedure. Upon emergence, the patient was agitated, restless, and moving around on the operation room table. He shouted with eyes closed, “Call a medic. I am hit. I am hit.” Additional staff was called in to prevent the patient from harming himself. He continued to yell for another five to seven minutes, at which point the patient was sedated with 70 mg of propofol. As the propofol wore off, the patient reemerged less agitated, but he remained disoriented. He continued to say, “I’m hit. I’m hit. Call a medic.” After another ten minutes, the patient’s spouse was gowned in disposable coveralls and brought into the operating room. The patient became calmer when he heard his wife's voice, but he remained disorientated for another two minutes. The moment of clarity came abruptly; he recognized his wife. The patient was again informed of his location and that surgery was completed. He immediately apologized and asked if he had hurt anyone.

### Case two

A 63-year-old male veteran presented for left carpal tunnel release and excision of lipoma of the left hand. His medical history included PTSD, bipolar disorder, anxiety, depression, obstructive sleep apnea, hypercholesterolemia, peripheral vascular disease, and chronic lower back and neck pain. His preoperative vital signs were stable, and his chronic conditions were all under control. For his anesthetic history, he reported having “violent wake-ups” from a colonoscopy as well as shoulder surgeries. He reported he sprained an ankle from a wild emergence from a colonoscopy a few months previously. Anesthesia plans and expected outcomes were discussed with the patient. The plan for verbal coaching using our voices was discussed as well. A 25-mcg infusion of dexmedetomidine given over 10 minutes was begun in the preoperative care unit. Upon arrival in the operation room and application of standard monitors, the patient was preoxygenated with 100% oxygen. He was induced with 100 mg of lidocaine, 200 mg of propofol, and 50 mcg of fentanyl given intravenously. A size four laryngeal mask airway was placed easily, and anesthesia depth was maintained with a lower one to 1.5% concentration of sevoflurane in an air/oxygen mixture with a fractional inspired oxygen concentration of 0.50. An additional 75 mcg of dexmedetomidine was given by infusion over 30 minutes. The surgeon injected 10 ml of 0.5% bupivacaine into the wound at closure. At the end of surgery, the patient was aroused by our verbal coaching while we controlled the noise levels in the operating room. He was able to verbalize the clarity of his understanding without agitation or disorientation while still in the operating room. He communicated with us in a calm manner while transferring to a recovery room.

## Discussion

### Speculation of the cases

The clinical presentations demonstrate the nature of ED in military veterans with PTSD. The content of their ED included agitation, aggression, high anxiety, and disorientation associated with their battlefield exposure. Although we suspected the patients’ anxiety levels associated with PTSD resulting from combat exposures might be a contributing factor for ED, we speculated the possible risk factors with these two cases.

Studies indicate the most common risk factors for ED after surgery are benzodiazepine use at preoperation and unmanaged postoperative pain [1-2]. We excluded using benzodiazepines for both cases and managed the surgical pain with opioids or dexmedetomidine. The different approaches for the two cases are reported in Table 1. Neither patient complained of pain after surgery, and no additional pain medications were required in the recovery room. Hypoxia and high oxygen supply were also reported to have a degree of relationship with the risk of developing ED [3]. Therefore, the patients were maintained with normal oxygen saturation on a fractional inspired oxygen concentration around 0.5 in an air and oxygen mixture. The surgeries were performed uneventfully, had minimal blood loss, and were completed in less than two hours. These factors, therefore, are excluded from the list of predisposing risk factors. Additionally, standardized anesthesia care and monitoring were used for both cases. When examining the patients' medical histories, neither showed any critical signs or symptoms that alerted the surgical team during the preoperative assessment. Thus, we excluded the possible contributing association between other comorbidities and the risk of ED occurrence. The common psychological illnesses both patients reported were anxiety and depression associated with diagnosed PTSD due to military combat exposures.

**Table 1:** Different anesthesia management for ED with PTSD in two cases

	Case One	Case Two
Preoperative management	No premedication	Initiated 25 mcg dexmedetomidine infusion over 20 minutes before induction Introduced a provider’s voice with voice coaching during emergence
Intraoperative management	Total 500 mcg of fentanyl and 1 mg of hydromorphone for pain management	Continued to infuse 75 mcg of dexmedetomidine over 30 minutes 50 mcg fentanyl at induction and no additional opioid required for pain management Local anesthetics by a surgeon Quiet surrounding during emergence Verbal coaching by the same anesthesia provider
Postoperative management	Verbal coaching Resedation with 75 mg of propofol intravenous injection Patient’s wife attenuated the symptoms of ED	No additional supports required
Outcome	ED	No ED

### ED with PTSD

The prevalence of ED in the general population was reported at 4.7% [1], while at 20% to 27% in combat veterans in the military population [4], PTSD was found to be significantly associated with both post-deployment physical and chronic mental health disorders [5]. Patients with PTSD were reported to experience flashbacks related to PTSD incidents, and the U.S. Department of Veterans Affairs reported 11% to 20% of combat veterans of Iraq and Afghanistan may undergo PTSD-related flashbacks for years [6].

### Dexmedetomidine and ED

Dexmedetomidine used in Case Two is a selective alpha 2-adrenergic receptor agonist and is known to have versatile characteristics that include sympatholytic, anxiolytic, sedative, anti-delirious, and analgesics. The benefits of using dexmedetomidine during cardiovascular surgeries by reducing postoperative death and myocardial complications were identified in multiple studies [6-7]. Maldonado and his associates reported patients sedated with dexmedetomidine had a lower chance of having postoperative delirium compared to those who received either propofol or midazolam [8]. Another experimental study found the effectiveness of sedation with dexmedetomidine for patients undergoing ventilator assistance or weaning from ventilator support in intensive care units [8-9]. Patients sedated with dexmedetomidine are reported to be easy to arouse but preserve a tranquil, sleep-like state without respiratory depression [10].

## Conclusions

This case report raises the unique characteristics of ED in military veterans with PTSD. Further studies are in great demand to fully understand the contributing factors associated with developing ED and possible solutions to reduce the risk of ED associated with PTSD among military veterans. Pharmacological, nonpharmacological, and multicomponent interventions should be considered to prevent ED in this population.

## References

[1] Lepousé C, Lautner CA, Liu L, Gomis P, Leon A (2006). Emergence delirium in adults in the post-anaesthesia care unit. Br J Anesth.

[2] Wofford K, Vacchiano C (2011). Sorting through the confusion: adverse cognitive change after surgery in adults. AANA J.

[3] Nollert G, Mohnle P, Tassani-Prell P (1995). Postoperative neuropsychological dysfunction and cerebral oxygenation during cardiac surgery. Thorac Cardiovasc Surg.

[4] McGuire JM (2012). The incidence of and risk factors for emergence delirium in U.S. military combat veterans. J Perianesth Nurs.

[5] Wachen JS, Shipherd JC, Suvak M, Vogt D, King LA, King DW (2013). Posttraumatic stress symptomatology as a mediator of the relationship between warzone exposure and physical health symptoms in men and women. J Trauma Stress.

[6] Flordellis C, Manolis A, Scheinin M, Paris Paris, H H (2004). Clinical and pharmacological significance of a2-adrenoceptor polymorphisms in cardiovascular diseases. Int J Cardiol.

[7] Talke P, Chen R, Thomas B (2000). The hemodynamic and adrenergic effects of perioperative dexmedetomidine infusion after vascular surgery. Anesth Analg.

[8] Maldonado JR, Wysong A, van der Starre PJ (2009). Dexmedetomidine and the reduction of postoperative delirium after cardiac surgery. Psychosomatics.

[9] Herr DL, Sum-Ping ST, England M (2003). ICU sedation after coronary artery bypass graft surgery: dexmedetomidine-based versus propofol-based sedation regimens. J Cardiothorac Vasc Anesth.

[10] Coull JT, Jones ME, Egan TD, Frith CD, Maze M (2004). Attentional effects of noradrenaline vary with arousal level: selective activation of thalamic pulvinar in humans. Neuroimage.

